# Complete plastome sequence of *Pseuderanthemum haikangense* C.Y. Wu & H.S. Lo (Acanthaceae): a medicinal plant in South China

**DOI:** 10.1080/23802359.2020.1810158

**Published:** 2020-08-25

**Authors:** Xu Gao, Hong-Xin Wang, Zhi-Xin Zhu, Hua-Feng Wang

**Affiliations:** Hainan Key Laboratory for Sustainable Utilization of Tropical Bioresources, College of Tropical Crops, Hainan University, Haikou, China

**Keywords:** *Pseuderanthemum haikangense*, plastome, phylogeny, genome structure, Acanthaceae

## Abstract

*Pseuderanthemum haikangense* (Acanthaceae) is a shrubs native to Guangdong, Hainan, Yunnan province of China. In this study, we report and characterize the complete plastome sequence of *P. haikangense* in order to provide genomic resources helpful for promoting its conservation and medicinal utilization. The complete plastome is 152,849 bp in length and contains the typical quadripartite structure of angiosperm, including two Inverted Repeat (IRs) regions of 25,849 bp, a Large Single-Copy (LSC) region of 83,878 bp and a Small Single-Copy (SSC) region of 17,273 bp. The plastome contains 113 genes, consisting of 79 unique protein-coding genes, 30 unique tRNA gene and 4 unique rRNA genes. The overall A/T content in the plastome of *P. haikangense* is 61.60%. The complete plastome sequence of *P. haikangense* will provide a useful resource for the conservation and garden utilization of this species as well as for the phylogenetic studies of Acanthaceae.

## Introduction

*Pseuderanthemum haikangense* C.Y. Wu & H.S. Lo is a shrub belonging to Acanthaceae. It is 40–100 cm tall. Its stem is terete, glabrous and the bark is straw-yellow. *P. haikangense* is native to Guangdong, Hainan, Yunnan province of China and distributed in forests with altitude from 200–900 m (Hu et al. 2011). It could be used as medicine to cure rheumatic paralysis. Consequently, the genetic and genomic information is urgently needed to promote its systematics research and the development of conservation value of *P. haikangense.* In this study, the complete plastome of *P. haikangense* (GenBank accession number: MT747169) was reported and characterized. This is the first report of a complete plastome for *P. haikangense.*

In this study, *P. haikangense* was sampled from Dahuajiao of Wanning city Nature Reserve in Hainan province of China (110.53°E, 18.79°N). A voucher specimen (Wang et al., B219) was deposited in the Herbarium of the Institute of Tropical Agriculture and Forestry (HUTB), Hainan University, Haikou, China.

The experiment procedure is as reported in Zhu et al. (2018). Around six Gb clean data were assembled against the plastome of *Echinacanthus lofouensis* (NC_035876.1) (Gao and Deng 2017) using MITObim v1.8 (Hahn et al. 2013). The plastome was annotated using Geneious R8.0.2 (Biomatters Ltd., Auckland, New Zealand) against the plastome of *E. lofouensis* (NC_035876.1).

The plastome of *P. haikangense* is found to possess a total length 152,849 bp with the typical quadripartite structure of angiosperms, contains two Inverted Repeats (IRs) of 25,849 bp, a Large Single-Copy (LSC) region of 83,878 bp and a Small Single-Copy (SSC) region of 17,273 bp. The plastome contains 113 genes, consisting of 79 unique protein-coding genes, 30 unique tRNA genes and 4 unique rRNA genes. The overall A/T content in the plastome of *P. haikangense* is 61.60%, which the corresponding value of the LSC, SSC and IR region were 63.50%, 67.20% and 56.70%, respectively.

**Figure 1.**
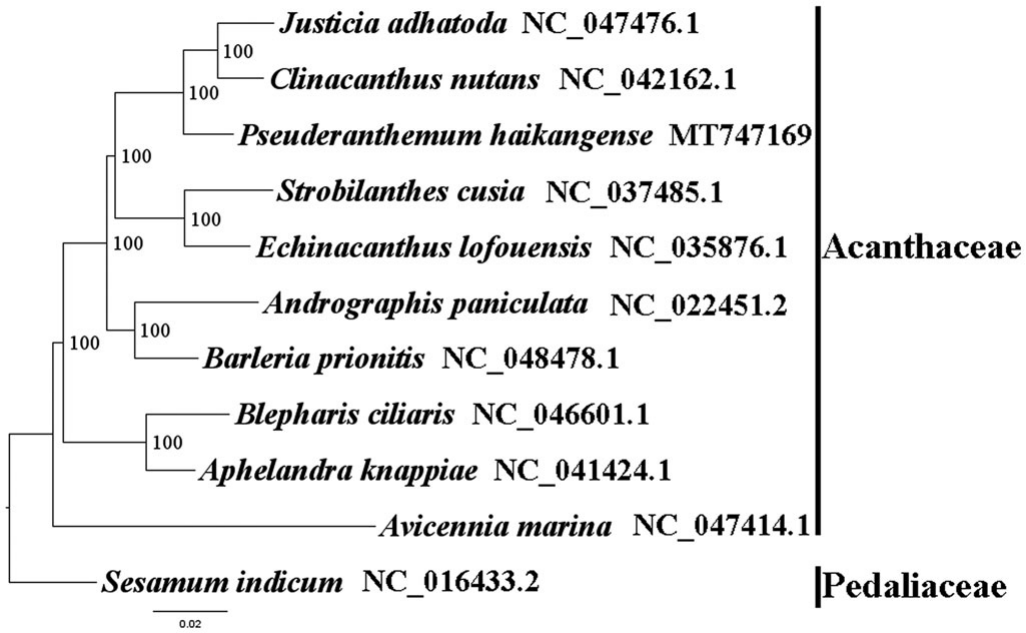
The best ML phylogeny recovered from 11 complete plastome sequences by RAxML. Accession numbers: *Pseuderanthemum haikangense* MT747169, *Justicia adhatoda* NC_047476.1, *Clinacanthus nutans* NC_042162.1, *Strobilanthes cusia* NC_037485.1, *Echinacanthus lofouensis* NC_035876.1, *Andrographis paniculata* NC_022451.2, *Barleria prionitis* NC_048478.1, *Blepharis ciliaris* NC_046601.1, *Aphelandra knappiae* NC_041424.1, *Avicennia marina* NC_047414.1. Outgroups: *Sesamum indicum* NC_016433.2.

We used RAxML (Stamatakis 2006) with 1000 bootstraps under the GTRGAMMAI substitution model to reconstruct a maximum likelihood (ML) phylogeny of nine published complete plastomes of Acanthaceae, using *Sesamum indicum* (Pedaliaceae) as an outgroup. The phylogenetic analysis indicated that *P. haikangense* is close to *Clinacanthus nutans* and *Justicia adhatoda* within Acanthaceae in this study (Figure 1). Most nodes in the plastome ML tree were strongly supported. The complete plastome sequence of *P. haikangense* will provide a useful resource for the conservation genetics and medicinal use of this species as well as for the phylogenetic studies of Acanthaceae.

## Data Availability

The data that support the findings of this study are openly available in GenBank of NCBI at http://www.ncbi.nlm.nih.gov, reference number MT747169.
